# DECIDER-2: Prospective randomized multicenter phase III trial of decitabine and venetoclax administered in combination with all-*trans* retinoic acid or placebo in patients with acute myeloid leukemia who are ineligible for induction chemotherapy

**DOI:** 10.1186/s12885-026-16617-8

**Published:** 2026-08-05

**Authors:** Olga Grishina, Claudia Schmoor, Caroline Sellner, Björn Hackanson, Jan-Henrik Mikesch, Iordanis Deligiannis, Felicitas Thol, Martina Crysandt, Christian Junghanss, Volker Runde, Paul La Rosée, Lino Lars Teichmann, Haifa Kathrin Al-Ali, Tim Sauer, Ulrich Germing, Frank Griesinger, Stefan Lukic, Jürgen Krauter, Snjezana Janjetovic, Jan Koch, Peter Staib, Julian Topaly, Maike de Wit, Doris Maria Kraemer, Lorenz Oelschläger, Ulf Schnetzke, Oliver Schmah, Swen Wessendorf, Cyrus Khandanpour, Lukas Kündgen, Monika Schwalenberg, Michael Heuser, Arnold Ganser, Hartmut Döhner, Ralph Wäsch, Michael Lübbert

**Affiliations:** 1 Clinical Trials Unit, Medical Center – University of Freiburg, Faculty of Medicine - University of Freiburg, Elsaesser Str. 2, Freiburg, 79110 Germany; 2 Department of Hematology/Oncology, Comprehensive Cancer Center Augsburg as Part of the CCC-WERA Consortium, University Medical Center Augsburg, Stenglinstraße 2, Augsburg, 86156 Germany; 3 Department of Medicine A, University Hospital Münster, Albert-Schweitzer-Campus 1, Münster, 48149 Germany; 4 Internal Medicine I, Brothers Hospital Trier, Nordallee 1, Trier, 54292 Germany; 5 Department of Hematology, Hemostasis, Oncology, and Cell Therapy, Hannover Medical School, Carl-Neuberg-Straße 1, Hannover, 30625 Germany; 6 Department of Hematology, Oncology, Hemostaseology and Stem Cell Transplantation, Medical Faculty, University Hospital Aachen, Pauwelsstraße 30, Aachen, 52074 Germany; 7 Department of Internal Medicine, Clinic for Hematology, Stem Cell Therapy and Palliative Medicine, Rostock University Medical Center, Ernst-Heydemann-Straße 6, Hemostasis, Rostock, Oncology 18055 Germany; 8 Department of Hematology, Oncology and Palliative Care, Wilhelm-Anton- Hospital, Voßheider Straße 214-232, Goch, 47574 Germany; 9 Department for Internal Medicine II, Schwarzwald‐Baar Klinikum, Klinikstraße 11, Villingen-Schwenningen, 78052 Germany; 10 Department of Medicine III, University Hospital Bonn, Venusberg-Campus 1, Bonn, 53127 Germany; 11 Krukenberg Cancer Center Halle, Medical Faculty, Martin-Luther- University Halle-Wittenberg, Ernst-Grube-Straße 40, Halle (Saale), 06120 Germany; 12 Department of Internal Medicine V, Heidelberg University Hospital, Im Neuenheimer Feld 410, Heidelberg, 69120 Germany; 13 Department of Hematology, Oncology, and Clinical Immunology, Heinrich- Heine University Düsseldorf, Moorenstraße 5, Düsseldorf, 40225 Germany; 14 Clinic for Hematology and Oncology, Pius-Hospital Oldenburg, University Medicine Oldenburg, Georgstraße 12, Oldenburg, 26121 Germany; 15 Department of Hematology and Oncology, Augusta-Kranken-Anstalt, Academic Teaching Hospital of the Ruhr-University Bochum, Bergstraße 26, Bochum, 44791 Germany; 16 Department of Hematology and Oncology, Municipal Hospital Braunschweig, Celler Straße 38, Braunschweig, 38114 Germany; 17 Department of Hematology and Cell Therapy, Helios Klinikum Berlin- Buch, Schwanebecker Chaussee 50, Berlin, 13125 Germany; 18 University Medical Center Mannheim (UMM), Theodor-Kutzer-Ufer 1-3, Mannheim, 68167 Germany; 19 Department of Hematology and Oncology, St Antonius Hospital Eschweiler, Dechant-Deckers-Straße 8, Eschweiler, 52249 Germany; 20 Department of Hematology and Oncology, CaritasKlinikum Saarbrücken, Rheinstraße 2, Saarbrücken, 66113 Germany; 21 Department of Internal Medicine, Hematology, Oncology and Palliative Medicine, Vivantes Klinikum Neukölln, Rudower Straße 48, Berlin, 12351 Germany; 22 St. Josefs-Hospital Hagen, Dreieckstraße 17, Hagen, 58097 Germany; 23 Department of Hematology and Oncology, Schleswig-Holstein, University Medical Center, University Cancer Center Schleswig-Holstein, Ratzeburger Allee 160, Lübeck, 23538 Germany; 24 Department of Hematology and Medical Oncology, Clinic for Internal Medicine II, Comprehensive Cancer Center Central Germany-Campus Jena, University Hospital Jena, Am Klinikum 1, Jena, 7747 Germany; 25 Section of Hematology and Oncology, Ortenau Medical Care Center, Ortenau Klinikum Lahr, Klostenstraße 19, Lahr, 77933 Germany; 26 Department of Hematology and Oncology, Klinikum Esslingen, Hirschlandstraße 97, Esslingen, 73730 Germany; 27 University Clinic for Internal Medicine – Oncology and Hematology, Oldenburg Medical Center, University of Oldenburg, Rahel-Straus-Straße 10, 26133 Oldenburg, Germany; 28 Department of Hematology, Oncology, Infectious Diseases and Palliative Care, Municipal Hospital Karlsruhe, Moltkestraße 90, Karlsruhe, 76133 Germany; 29 Department of Hematology, Oncology and Palliative Care, Hospital Lüdenscheid, Paulmannshöher Str. 14, Lüdenscheid, 58515 Germany; 30 Department of Internal Medicine IV, University Hospital Halle (Saale), Martin-Luther-University Halle-Wittenberg, Ernst-Grube-Str. 40, Halle (Saale), 06120 Germany; 31 Department of Internal Medicine III, University Hospital Ulm, Albert-Einstein-Allee 23, Ulm, 89081 Germany; 32 Department of Hematology, Oncology and Stem Cell Transplantation, Medical Center – University of Freiburg, Faculty of Medicine - University of Freiburg, Hugstetter Straße 55, Freiburg, 79106 Germany

**Keywords:** Acute myeloid leukemia, All-*trans* retinoic acid, Elderly patients, Low-dose decitabine, Venetoclax

## Abstract

**Background:**

Acute myeloid leukemia (AML) is predominantly a disease of older patients with a poor long-term survival. Approval of the combination of venetoclax (VEN) with azacitidine (AZA) or decitabine (DAC) in the European Union in 2021 for the treatment of patients with newly diagnosed AML who are ineligible for induction chemotherapy has become the treatment backbone for older, medically non-fit AML patients. Based on in vitro priming of AML cells to all-*trans* retinoic acid (ATRA) by DAC, we had previously conducted a randomized phase II trial (DECIDER, AMLSG 14 − 09) in 200 elderly, non-fit AML patients on the effect of ATRA as add-on to DAC. Median overall survival (OS) was 8.2 months with ATRA versus 5.1 months without ATRA (hazard ratio, 0.65; 95% CI, 0.48 to 0.89; *P* = 0.006). Notably, ATRA did not add toxicity, the combination was active also in patients with adverse genetics, and prolonged time to treatment resistance. Investigating a triple combination of DAC, VEN and ATRA was a logical next step. This randomized double-blind phase III trial compares the efficacy of ATRA versus placebo as add-on to the backbone treatment (DAC and VEN) with respect to OS, objective best response, quality of life and safety. The accompanying translational research will contribute to identify molecular markers for drug efficacy and better tailoring epigenetic therapy.

**Methods:**

DECIDER-2 (AMLSG 32 − 21) is a prospective, randomized, double-blind, placebo-controlled, parallel group, multicenter trial. The primary study objective is to compare the efficacy of ATRA versus placebo as add-on to the backbone treatment (DAC and VEN) in terms of OS. The target population is adult AML patients unfit for standard induction chemotherapy. Patients are randomized (1:1) to receive backbone treatment in combination with either ATRA or placebo. Four interim safety analyses were planned with the involvement of the Data Monitoring Committee.

**Discussion:**

This trial investigates whether the in vitro cooperativity of DAC, VEN, and ATRA prolongs OS in unfit AML patients compared to placebo, offering a novel, low-toxicity triplet combination with a promising risk-benefit ratio.

**Clinical trial number:**

EU CT number: 2020-005495-36/2023-507461-26-00 (registered 18.11.2020); German clinical trials registry number: DRKS00023646 (registered 23.08.2022).

## Background

Acute myeloid leukemia (AML) is predominantly a disease of older patients, with a median age of > 65 years [1] who, however, are underrepresented in trials of standard induction chemotherapy. Ineligibility for intensive treatment is often due to reduced performance status, comorbidities etc [2].

Present treatment options for elderly AML patients unfit for standard chemotherapy are based on single-agent hypomethylating agents (HMAs) [3, 4], low- dose cytarabine, and more recently, HMA in combination with venetoclax (VEN) [5]. For unfit AML patients with mutations of the *IDH1/2* gene, specific inhibitors have been recently approved or integrated in clinical practice.

The research question arising from the medical problem addressed in the trial is whether an in vitro cooperativity between decitabine (DAC), VEN and ATRA can be confirmed in vivo, with extension of overall survival (OS) of AML patients receiving this 3-drug combination when compared to DAC, VEN and placebo. This triplet combination is novel and, since ATRA has very limited toxicities, promising regarding the risk-benefit ratio when administered to medically non-fit patients, in whom toxicity issues need to be particularly carefully considered during trial planning.

The impact of the results of this study is directly related to extension of OS of AML patients. Beyond AML treatment, a better understanding of in vivo cooperativity between HMAs and ATRA is potentially highly relevant also for other malignant disorders including solid tumors.

### Evidence and need of the trial

Our previous randomized clinical trial DECIDER (for DECItabine, DEacetylase inhibition, Retinoid) was conducted at 27 German trial centres and recruited 200 patients. The trial had a 2 × 2 design investigating the effects of ATRA and the HDAC inhibitor valproate as add-on to DAC. Patients were randomized to the following four treatment arms: DAC (*n* = 47), DAC+valproate (*n* = 57), DAC+ATRA (*n* = 46), and DAC+valproate+ATRA (*n* = 50).

DECIDER was a phase II study with primary endpoint objective best response (complete and partial remission) for a first efficacy assessment of valproate and ATRA add-on. For valproate, no effect was observed with respect to objective best response and overall survival. A beneficial effect of ATRA vs. no ATRA was present with respect to both best response and overall survival. The 1-year overall survival rates were 35% vs. 20%, median overall survival times were 8.2 vs. 5.1 months, hazard ratio (HR) = 0.65, 95% confidence interval (CI) [0.48,0.88], *p* = 0.006, ATRA vs. no ATRA, respectively [6].

The clinical benefit with ATRA was not at the expense of increased toxicity, in line with the safety concerns when combining drugs to treat elderly AML patients. Notably, patients receiving ATRA had a longer time to resistance development, and the survival benefit observed was similar in intermediate and adverse genetic risk groups. Following the positive results of the DECIDER trial, we planned to confirm this effect of ATRA in a placebo-controlled phase III clinical trial. Given the very impressive results in newly diagnosed AML patients receiving VEN in combination with AZA or DAC (see below), we hypothesized that when ATRA is combined with VEN and DAC in a “triplet”, response duration may be prolonged compared to the dual treatment with VEN and DAC, potentially via delay of development of secondary resistance.

In recent years, the treatment of AML patients ineligible for standard induction chemotherapy (IC), has made unexpected strides regarding the rapid and broad acceptance of the dual combination of a DNA hypomethylating agent (HMA) and the *BCL-2* inhibitor VEN. The VIALE-A phase III trial [5] demonstrated that the HMA azacitidine (AZA) plus VEN is superior to AZA monotherapy in most AML subgroups but not in *TP53* mutated patients [7]. HMA + VEN treatment has also been effective as „bridging“ approach in AML patients fit for the curative approach of allografting [8, 9]. This is not surprising considering the more favorable safety profile of HMA-based treatment compared to IC, as demonstrated in the randomized phase III Intergroup study AML21, coordinated by the EORTC Leukemia Group that compared an extended, 10-day DAC monotherapy induction to standard „7 + 3“ induction in AML patients aged 60 years and older [10, 11]. Very recently, DAC + VEN has been investigated, in a randomized phase II trial conducted by a Chinese consortium, as an alternative to intensive chemotherapy (idarubicin and cytarabine) in newly diagnosed AML patients aged 18–59 years, exhibiting a non-inferior response rate, but with fewer SAEs [12]. Indeed, the rapid and general adoption of an HMA-VEN de-escalation approach has prompted the initiation of multiple studies investigating triple combinations, e.g. HMA-VEN combined with inhibitors of *FLT3* [13], *IDH1/2* [14] or menin [15], a strategy that is similar to the approach we had taken in the DECIDER-2 trial as well as its predecessor DECIDER (DAC/valproic acid/ATRA).

Also in recent years, the potential of an HMA combined with ATRA has garnered renewed interest (reviewed by Geoffroy et al., 2021 [16]). In preclinical work we could show that in AML cell lines, DAC and ATRA had cooperative effects on induction of apoptosis and differentiation compared to either treatment alone; furthermore cells with mutated *TP53* treated with DAC+ATRA disclosed more effective derepression of transposable elements („viral mimicry“) than those with WT TP53 [17]. Similarly, the group of Prof. Hongyan Tong (Hangzhou, China) showed that ATRA enhances the cytotoxic effects of DAC in AML blasts by activating the RARα-Nrf2 complex [18]. El Khawanky et al. (2025) [19] demonstrated that AZA efficacy, which is modulated by intrinsic RNA receptor signalling, can be restored by ATRA during acquired resistance. In a post-hoc subgroup analysis of patients from the DECIDER trial, we asked whether TP53-mutated patients also benefit from the HMA+ATRA combination; indeed, in patients with TP53 mutations the response rate in those receiving DAC+ATRA was higher when compared to those not receiving the ATRA add-on [20]. The survival benefit of TP53-mutated patients mediated by ATRA add-on was reduced, but not completely negated, warranting further investigation. Clinical evidence for efficacy of the HMA+ATRA combination was also generated in MDS: a randomized phase 2 trial in MDS-EB patients, coordinated by the Hangzhou group, also addressed the question of the benefit of ATRA add-on to DAC. The study resulted in a significantly higher response rate and progression-free survival in patients receiving the combination, compared to DAC monotherapy [21].

In conclusion, the evidence of preclinical and clinical studies generated in recent years supports the underlying hypothesis that a triplet of DAC, VEN and ATRA may be superior to DAC + VEN in AML patients of different risk groups, potentially including also TP53 mutated patients.

## Methods/design

### Study design and setting

This is a prospective, randomized, double-blind, placebo-controlled, parallel group, multicenter phase III trial. The primary objective of the trial is to compare the efficacy of all-*trans* retinoic acid (ATRA) versus placebo as add-on to the backbone treatment (DAC + VEN) in older and unfit AML patients with respect to OS. Secondary objectives are to compare ATRA *versus* placebo as add-on to the backbone treatment (DAC + VEN) with respect to objective best response, i.e. CR/CRi/MLFS/PR, CR with negative measurable residual disease (MRD), probability of survival with objective best response, quality of life (especially fatigue) and safety.

Trial sites are located in Aachen, Augsburg, Berlin-Buch, Berlin-Neukölln, Bochum, Bonn, Braunschweig, Düsseldorf, Eschweiler, Esslingen, Freiburg, Hagen, Halle, Hannover, Heidelberg, Jena, Karlsruhe, Kleve-Goch, Lahr, Lübeck, Lüdenscheid, Mannheim, Münster, Oldenburg, Rostock, Saarbrücken, Trier, Ulm and Villingen-Schwenningen.

The study received approval from the competent authority and ethics committees of the University of Freiburg as well as the respective local ethics committees and was successfully submitted to the CTIS. Patient’s written consent to participate in this clinical trial and translational research program was obtained prior to any study-specific procedures. The identifying number of the DECIDER-2 trial in the German clinical trials registry is DRKS00023646 and in EU clinical trials register 2020-005495-36/2023-507461-26-00.

### Study population

The target population of this trial represents patients older than 18 years with AML according to WHO (≥ 20% blasts in the peripheral blood or bone marrow) not qualifying for, or not consenting to, standard remission-induction chemotherapy or immediate allografting. Key inclusion criteria are: patient’s written informed consent; 18 years or older at time of informed consent; patients with primary or secondary AML according to WHO who are not expected to benefit from standard remission-induction chemotherapy; <25.000 leukocytes/µl and performance status ECOG 0, 1, 2. Key exclusion criteria are: Acute promyelocytic leukemia (APL), AML of FAB subtype M3; previous remission-induction chemotherapy for MDS or AML; previous allogeneic stem cell transplantation; previous treatment with DAC, 5-azacytidine, VPA or other DNA-hypomethylating agents, ATRA, venetoclax and other Bcl-2 inhibitors; “low-dose” chemotherapy (e.g. hydroxyurea, cytarabine (Ara-C), melphalan etc.) within four weeks prior to the first ATRA/placebo administration, except for cytoreduction of leukocytosis ≥ 25.000/µl with hydroxyurea or Ara-C as prescribed by the study protocol. Further exclusion criteria are related to general medical conditions or contraindications to administration of investigational product according to Summary of Product Characteristics.

### Study treatment and procedures

**Fig. 1:**
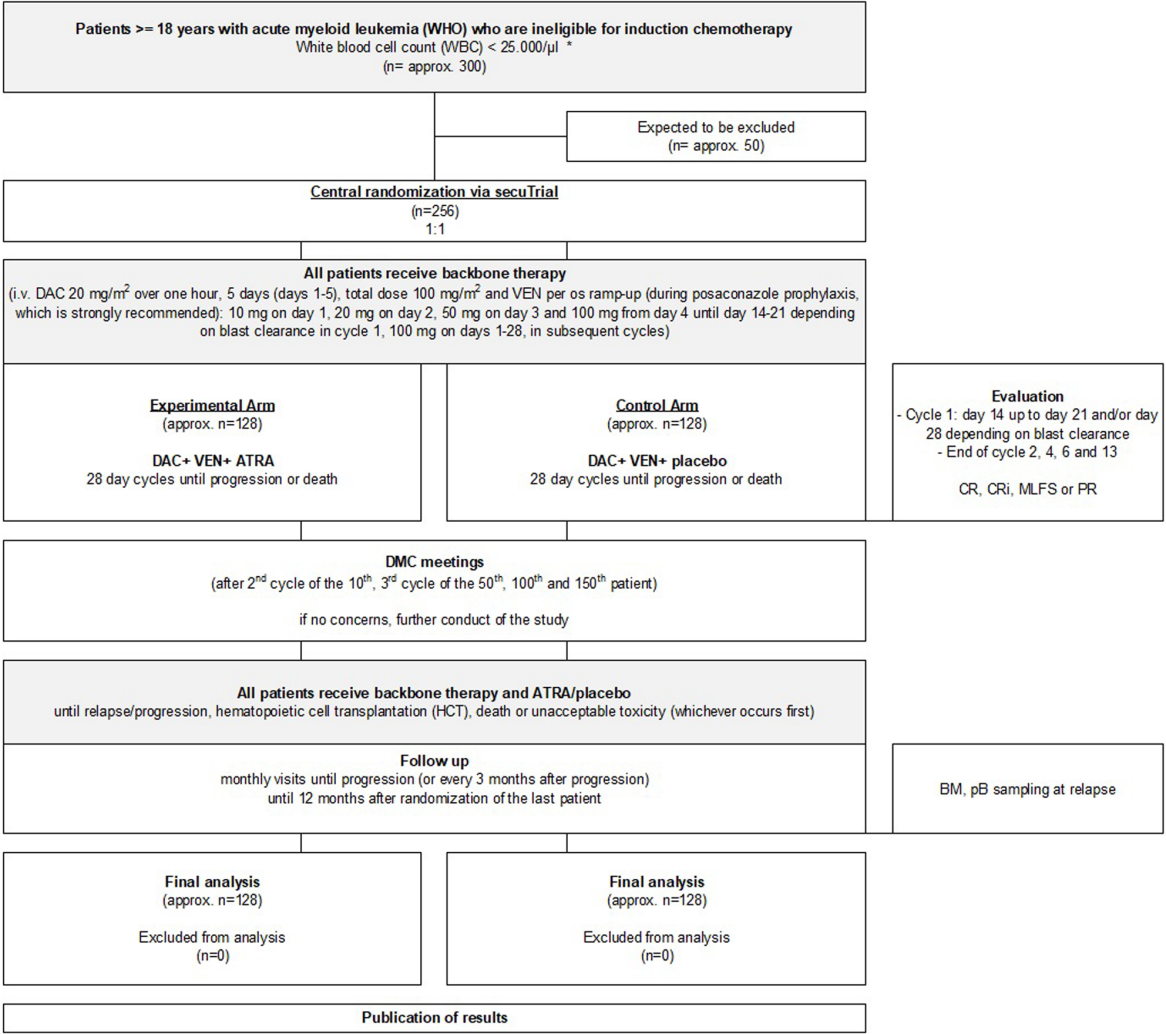
Trial Flow. *If WBC ≥ 25.000/µl: Hydroxyurea (HU) or Ara-C until WBC < 25.000/µl. ATRA = all-*trans* retinoic acid; BM = bone marrow; CR = complete remission; CRi = complete remission with incomplete count recovery; DAC = decitabine; DMC = data monitoring committee; MLFS = morphologic leukemia-free state; pB = peripheral blood; PR = partial remission; VEN = venetoclax; WBC = white blood cell count; WHO = world health organization

Study treatment in the two study arms (Fig. 1) is as follows. Experimental Arm: backbone treatment (consisting of intravenous DAC 20 mg/m² over 1 h, 5 days [total dose 100 mg/m²], repeated every 4 weeks, and VEN *per os* (p.o.) ramp-up (during posaconazole prophylaxis, which is strongly recommended): 10 mg on day 1, 20 mg on day 2, 50 mg on day 3 and 100 mg from day 4 until day 14 or 21/28 depending on blast clearance), and ATRA p.o. 45 mg/m^2^ days 6–28. Control Arm: backbone treatment and placebo p.o. corresponding to ATRA dose.

Treatment cycles (backbone and study medication) are repeated until relapse/progression, hematopoietic cell transplantation (HCT), death or unacceptable toxicity (whichever occurs first). Response assessment is performed in cycle 1, at the end of the cycles 2, 4, 6, and 13 and in case of suspected relapse/progression. Study visits are carried out at least once a month during the treatment and every three months after treatment discontinuation until 12 months after randomization of the last patient.

### Study endpoints

The primary endpoint is the OS defined as time from the date of randomization to the date of death from any cause. Secondary endpoints for the evaluation of efficacy are the following: (1) objective best response (OBR) defined as CR, CRi, morphologic leukemia-free state (MLFS) or partial remission (PR) assessed after cycles 1, 2, 4, 6, and 13. Patients are counted as objective responders if they achieve a CR, CRi, MLFS or PR. All other patients are counted as non-responders. The determination of CR, CRi, MLFS or PR is performed according to the standard criteria [22]; (2) response defined as CR with negative measurable residual disease (CR_MRD-_) assessed after cycles 2, 4, and 6; (3) survival probability after attainment of objective best response (CR, CRi, MLFS or PR) defined as time from objective best response to the date of death from any cause; (4) quality of life (QOL) of the patients using the EORTC QLQ-C30 Quality of Life Questionnaire, and FACIT Fatigue Scale. Further secondary endpoint for evaluation of the treatments is safety and toxicity, which are evaluated by means of adverse events (AEs) occurring from the first administration until 30 days after the last administration of study medication or first day of hospitalization for HCT or until start date of any tumor-specific treatment in case of relapse/progression (whichever occurs first) and serious AEs related to the study medication as per investigator’s judgment reported until end of the study.

### Sample size

Sample size calculation is based on the primary endpoint OS. The study should have a power of 80% to demonstrate superiority of ATRA vs. placebo with respect to OS at one-sided level alpha of 2.5% under the assumption of a hazard ratio of 0.67. This requires the observation of 191 deaths. A hazard ratio of 0.67 would correspond e.g. to a median OS time of 12 months vs. 8 months. The median OS time of 286 patients treated with AZA plus VEN 400 mg in the VIALE-A trial by DiNardo et al. (2020) [5] was reported as 14.7 months, in the control group of 145 patients treated with AZA plus placebo it was reported as 9.6 months (hazard ratio 0.66, 95% confidence interval [0.52, 0.85], *p* < 0.001 of VEN vs. placebo). In the DECIDER trial, the median OS time of 96 patients treated with DAC+ATRA (+/- valproate) was 8.2 months, and of 104 patients treated with DAC (+/-valproate) was 5.1 months, with a hazard ratio of 0.65, 95% confidence interval [0.48,0.88], *p* = 0.006 of ATRA vs. no ATRA [6].

Thus, the assumption of median OS times of 12 vs. 8 months for the planned trial seems to be realistic. Under this assumption, and a planned recruitment period of 3 years and an additional follow-up period of 1 year, it is necessary to include 230 patients (nQuery Advisor 7.0). If longer median OS times were observed (e.g. 15 vs. 10 months, also corresponding to a hazard ratio of 0.67), the follow-up period had to be extended to 1.5 years, in order to observe the required number of 191 deaths with the same number of included patients. To account for the possibility of up to 10% of the patients with incomplete follow-up, 230/0.9 = 256 patients will be randomized in total (ratio 1:1).

### Randomization

The randomization code is generated by the CTU ensuring that treatment assignment is unbiased and concealed from patients and investigator staff. Randomization is performed, stratified by site and by sex, in blocks of variable length in a ratio of 1:1. The block lengths are documented separately and will not be disclosed to the sites. The randomization code is produced by validated programs based on the Statistical Analysis System (SAS) and inserted into the secuTrial^®^ system.

### Statistical analysis

The primary estimand that corresponds to the primary trial objective “comparison of the efficacy of ATRA versus placebo as add-on to the backbone treatment DAC + VEN with respect to OS” is specified by the following 5 attributes (according to ICH E9 (R1)).

Population: Newly diagnosed AML patients as defined by the before mentioned inclusion and exclusion criteria randomized to one of the treatment arms (experimental vs. control) who received at least one dose of study treatment. The population will be called full analysis set (FAS).

Treatment: The randomized experimental arm backbone treatment DAC + VEN and IMP ATRA is compared to the randomized control arm backbone treatment DAC + VEN and Placebo regardless of dosing modifications, treatment interruptions, treatment discontinuation, regardless of intake of additional medications, and regardless of treatment with HCT.

Endpoint: OS defined as time from the date of randomization to the date of death from any cause. For patients surviving the time under observation in the trial, the time from the date of randomization to the date of the last follow up (visit or phone call) will be analyzed as censored observation.

Intercurrent events: Dosing modifications, treatment interruptions, discontinuation of the study treatment, intake of additional medications, treatment with HCT will be ignored. This corresponds to the treatment policy strategy according to ICH E9 (R1).

Population-level summary: The population-level summary is defined by the following statistical analysis approach. A Cox regression model being stratified for site and including, in addition to the effect of ATRA, sex as covariate for adjustment will be used. As estimate of the effect size, the hazard ratio (ATRA vs. placebo) will be calculated with corresponding two-sided 95% confidence interval. Superiority of ATRA vs. placebo will be tested from this model using the Wald test at one-sided level alpha of 2.5%. OS rates will be estimated by the Kaplan-Meier method by randomized treatment.

The estimands corresponding to the secondary trial objectives and endpoints are specified accordingly in the clinical trial protocol. The analysis of the treatment effects with respect to objective best response (CR, CRi, MLFS or PR) and with respect to CR_MRD-_ at least at one of the time points (after cycles 2, 4, or 6) will be performed with logistic regression models including, in addition to the effect of ATRA, site and sex as covariates for adjustment. As estimate of the effect sizes, the odds ratios (ATRA vs. placebo) will be calculated with corresponding two-sided 95% confidence intervals.

For evaluating QOL, EORTC QLQ-C30 scores will be calculated according to Fayers et al., 2001 [23], and the FACIT Fatigue scale will be calculated according to FACIT Administration and Scoring Guidelines [24]. At the different time points during follow-up changes in relation to baseline will be calculated. With respect to the secondary endpoints QOL, the treatment groups will be compared descriptively.

Safety is analyzed in the safety population including all randomized patients who received at least one dose of study medication, and according to the received treatment. AEs are coded using Medical Dictionary for Regulatory Activities (MedDRA). The probability of AEs defined by preferred term (PT) according to MedDRA and of AEs defined by system organ class (SOC) according to MedDRA will be estimated by the Aalen-Johansen estimator. For this purpose, the time from start of the first ATRA/placebo intake to the first occurrence of the specific type of AE (defined by PT or defined by SOC) will be analyzed. For patients not experiencing the specific type of AE, the time to the end of the documentation period or death, whatever occurs first, will enter the analysis and will be treated as a competing event. This will result in probability estimates (cumulative incidences) for the different AE types (defined by PT and by SOC) over time. The results will be displayed in summary tables by showing the cumulative incidences at the maximum observation time.

### Interim analysis

No efficacy interim analyses will be performed. The first interim safety analysis was performed after the 2nd cycle of the 10th randomized patient. Thereafter the interim analyses were done after the 3rd cycle (84 days) of the 50th, the 100th and the 150th randomized patient. The safety analyses were performed as soon as possible after the respective data became available; as planned, patient recruitment was not interrupted.

The objective of these analyses is to enable stopping or modification of treatments and procedures in case of unacceptable safety or death rates. Since these analyses cannot lead to the conclusion of superiority of ATRA vs. placebo, no alpha adjustment in the final analysis is necessary.

### Central cytogenetics and molecular diagnostics

Central cytogenetics and molecular diagnostics are being performed in central the reference laboratory of the German–Austrian Acute Myeloid Leukemia Study Group (AMLSG), at the University of Ulm, or at another reference center selected by the site.

For conventional cytogenetic analysis, standard techniques are used and chromosomal abnormalities are described according to the International System for Cytogenetic Nomenclature. The adherence in sending samples to the central laboratory is about 90% of the patients. Molecular genetic characterisation of patient samples is performed according to the European LeukemiaNet Recommendations [22] and include screening for at least the following gene mutations: *NPM1*,* CEBPA*,* FLT3*,* ASXL1*,* RUNX1*,* TP53* and gene rearrangements: *PML::RARA*,* CBFB::MYH11*,* RUNX1::RUNX1T1*,* BCR::ABL1*.

### Central measurable residual disease (MRD) assessment

MRD assessment is carried out at the central laboratory of University Hospital Halle, Internal Medicine IV (Hematology and Oncology), Halle (Saale). The flow cytometric quantification of the MRD at screening and during the first six treatment cycles is performed using multi-color flow cytometry following a standardized protocol of the ELN MRD working group [25].

### Central Pharmacy

The central pharmacy of the University Medical Center Mainz has a long-standing experience in the preparation of study medication, including placebo. Its task is to manufacture and distribute the oral ATRA/placebo capsules to the study sites in accordance with German laws, Good Manufacturing and Good Distribution Practice.

### Translational research program

The study provides the opportunity to address not only baseline genetic and epigenetic characteristics of the patient blasts with relation to the clinical response, but also mRNA expression and epigenetic changes induced by the treatment. Bone marrow and peripheral blood cells are procured prior to treatment within the standard diagnostic workup, and in patients consenting to serial sampling, also at defined, early and late time points during the treatment.

### Quality assurance system

During the clinical trial, quality control is ensured through monitoring, auditing and supervision by the authorities, if applicable. The Clinical Trials Unit of the Medical Center - University of Freiburg, Germany, is responsible for project coordination, statistical planning and analysis, data management, clinical monitoring and pharmacovigilance. An independent DMC consisting of three hemato-oncologists and one statistician was established. The function of the DMC is to monitor and supervise the progress of the trial (including safety data and adherence to the protocol) and to advise whether to continue, modify or stop the trial. Composition and responsibilities of the DMC, structure and content of its meetings, and its relationship to other key study team members are laid down in a separate DMC charter. The DMC members are continuously informed about study progress and safety data. The DMC gave recommendations on study conduct after the review of the extensive reports about the interim safety analyses including data on patient recruitment, baseline characteristics, eligibility violations, treatment compliance, and safety separately for the different treatment arms. These analyses were conducted by an independent statistician. The study team remained blinded.

### Study status

Since the start of recruitment in October 2022, a total of 233 patients were randomized in the study until June 16, 2026. Four safety interim analyses were conducted in October 2023, October 2024, September 2025 and April 2026 by the Clinical Trials Unit; the confidential blinded reports were made available only to the coordinating investigator, medical coordinator, and to the DMC. The confidential unblinded reports were made available only to the DMC. Following the DMC recommendation a routine documentation of available platelet and absolute neutrophil count (ANC) was implemented to better monitor hematologic reconstitution. No important safety issues have been observed that would alter the assumed benefit-risk profile. The DMC statements released shortly after corresponding meetings recommended continuing the study as planned, as all DMC members unanimously agreed that at the moment there are no ethical or other concerns on further conduct of the trial.

## Discussion

Until recently, treatment options for elderly AML patients were very limited because they are less capable of tolerating intensive cytotoxic induction and post-remission chemotherapy. The research question addressed in the proposed trial is whether an in vitro cooperativity between DAC, VEN and ATRA can be confirmed in vivo, with extension of overall survival of AML patients receiving this 3-drug combination when compared to DAC, VEN and placebo. This triplet combination is novel and, since ATRA has very limited toxicities, promising regarding the risk-benefit ratio when administered to medically non-fit patients, in whom toxicity issues need to be particularly carefully considered during trial planning. Thus, the aim of our trial is to investigate the potential benefit of addition of ATRA to DAC and VEN with regard to overall survival and disease control in a randomized double-blind setting. As recommended by the DMC, after examination of the patients’ data in frame of the interim safety analyses, the study will be continued as initially planned.

## Data Availability

No datasets were generated or analysed during the current study.

## Abbreviations

AE: Adverse event
AML: Acute myeloid leukemia
AMLSG: German–austrian acute myeloid leukemia study group
ATRA: All-trans retinoic acid
AZA: azacitidine
CR: Complete remission
CRi: Complete remission with incomplete count recovery
CTU: Clinical trials unit, Freiburg
DAC: Decitabine
DMC: Data monitoring committee
EORTC: European organization for research and treatment of cancer
HDAC: Histone deacetylases
HMA: Hypomethylating agent
MDS: Myelodysplastic syndrome
MLFS: Morphologic leukemia-free state
MRD: Measurable residual disease
OS: Overall survival
p.o.: per os
PR: Partial remission
QOL: Quality of life
SD: Stable disease
VEN: Venetoclax
Vs.: versus
WBC: White blood cell count
WHO: World health organization
